# Novel two‐lead cardiac resynchronization therapy system provides equivalent CRT responses with less complications than a conventional three‐lead system: Results from the QP ExCELs lead registry

**DOI:** 10.1111/jce.14552

**Published:** 2020-06-01

**Authors:** Naushad A. Shaik, Michael Drucker, Christopher Pierce, Gabor Z. Duray, Shane Gillett, Crystal Miller, Camden Harrell, George Thomas

**Affiliations:** ^1^ Department of Cardiac Electrophysiology Advent Health Orlando Orlando Florida; ^2^ Department of Cardiac Electrophysiology Novant Health Cardiology of Forsyth Medical Center Winston‐Salem North Carolina; ^3^ Department of Cardiac Electrophysiology Sanford Medical Center Fargo North Dakota; ^4^ Department of Cardiology, Medical Centre Hungarian Defense Forces Budapest Hungary; ^5^ Clinical Studies Department Biotronik, Inc Lake Oswego Oregon; ^6^ Division of Cardiology Weill Cornell Medical College New York New York

**Keywords:** atrial fibrillation, atrial sensing, cardiac resynchronization therapy, heart failure, implantable cardioverter‐defibrillator

## Abstract

**Introduction:**

The novel two‐lead cardiac resynchronization therapy (CRT)‐DX system utilizes a floating atrial dipole on the implantable cardioverter‐defibrillator lead, and when implanted with a left ventricular (LV) lead, offers a two‐lead CRT system with AV synchrony. This study compared complication rates and CRT response among subjects implanted with a two‐lead CRT‐DX system to those subjects implanted with a standard three‐lead CRT‐D system.

**Methods and Results:**

A total of 240 subjects from the Sentus QP—Extended CRT Evaluation with Quadripolar Left Ventricular Leads postapproval study were selected to identify 120 matched pairs based on similar demographic characteristics using a Greedy algorithm. The complication‐free rate was evaluated as the primary endpoint. All‐cause mortality, heart failure hospitalizations, device diagnostic data, New York Heart Association (NYHA) class improvement, and defibrillator therapy were evaluated from clinical data, in‐office interrogations, and remote monitoring throughout the follow‐up period. Complication‐free survival favored the CRT‐DX group with 92.5% without a major complication compared to 85.0% in the CRT‐D cohort (*P* = .0495; 95% confidence interval: 0.1%‐14.9%) over a mean follow‐up of 1.3 and 1.4 years, respectively. Incidence of all‐cause mortality, heart failure hospitalizations, NYHA changes at 6 months postimplant, and percent of LV pacing during CRT therapy were similar in both device cohorts. Inappropriate shocks were more frequent in the CRT‐D cohort with 5.8% of subjects receiving an inappropriate shock vs 0.8% in the CRT‐DX cohort.

**Conclusion:**

The results of this subanalysis demonstrate that the CRT‐DX system can provide similar CRT responses and significantly fewer complications when compared to a similar cohort with a conventional three‐lead CRT‐D system.

## 1 INTRODUCTION

Cardiac resynchronization therapy (CRT) is a proven treatment to reduce morbidity and mortality in patients with heart failure.^1^, ^2^, ^3^ Compared to single or dual‐chamber implantable cardioverter‐defibrillator (ICD) therapy, risks associated with CRT can include increased complication rates.^4^, ^5^, ^6^, ^7^ The DX system can deliver CRT utilizing a novel two‐lead system that incorporates atrial sensing on the ICD lead for SVT discrimination and AV synchronization, thus allowing for CRT delivery with fewer leads. The two‐lead CRT‐DX systems are considered for subjects without a need for atrial pacing, including those with permanent atrial fibrillation (AF).

Prior studies have demonstrated the accuracy and utility of atrial sensing with an ICD lead with a floating atrial dipole.^8^, ^9^, ^10^, ^11^, ^12^ Additionally, several studies have compared the complication rates in conventional three‐lead CRT‐D systems to those rates seen in dual and single chamber ICD systems.^4^, ^5^, ^6^, ^7^ However, no studies have evaluated the complication rates in a population of heart failure patients implanted with a two‐lead CRT‐DX system, and only one prior study exists to compare two‐lead CRT‐DX and three‐lead CRT‐D system performance.^13^

This subanalysis of the Sentus QP—Extended CRT Evaluation with Quadripolar Left Ventricular Leads (QP ExCELs) study compares the complication rates and CRT response of subjects implanted with two‐lead CRT‐DX systems to those subjects implanted with conventional three‐lead CRT‐D systems.

## 2 METHODS

We retrospectively analyzed complication rates and CRT responses from subjects who were enrolled in the QP ExCELs study at US sites. QP ExCELs is a prospective, multicenter, international, nonrandomized, combined premarket study, and postapproval registry which enrolled 1907 US patients to evaluate the safety and efficacy of the Sentus QP left ventricular (LV) lead (BIOTRONIK SE & Co KG, Berlin, Germany) through 5 years postimplant (Clinicaltrials.gov Identifier: NCT02290028). The study was approved by the institutional review board at each of the 74 US participating sites. Potential patients were identified by the investigator from their general patient population and patients provided written informed consent before study procedures. All subjects included in the QP ExCELs study had a standard CRT‐D indication.

CRT‐DX systems consist of a CRT‐DX pulse generator and a DX ICD lead. The DX ICD lead is a 7.8 French, bipolar, single coil, active fixation ICD lead. Two electrodes spaced 15 mm apart and mounted 15 to 17 cm proximal to the lead tip comprise a larger atrial sensing dipole to provide atrial diagnostics and, therefore, eliminate the need for an atrial lead for effective atrial sensing. The atrial signal is amplified up to four times and bandpass‐filtered to enable P‐wave detection while excluding signal frequencies outside of the atrial range.^14^, ^15^ The SMART detection algorithm available in the DX system utilizes the atrial signal to provide SVT discrimination.^8^, ^14^

**Figure 1:**
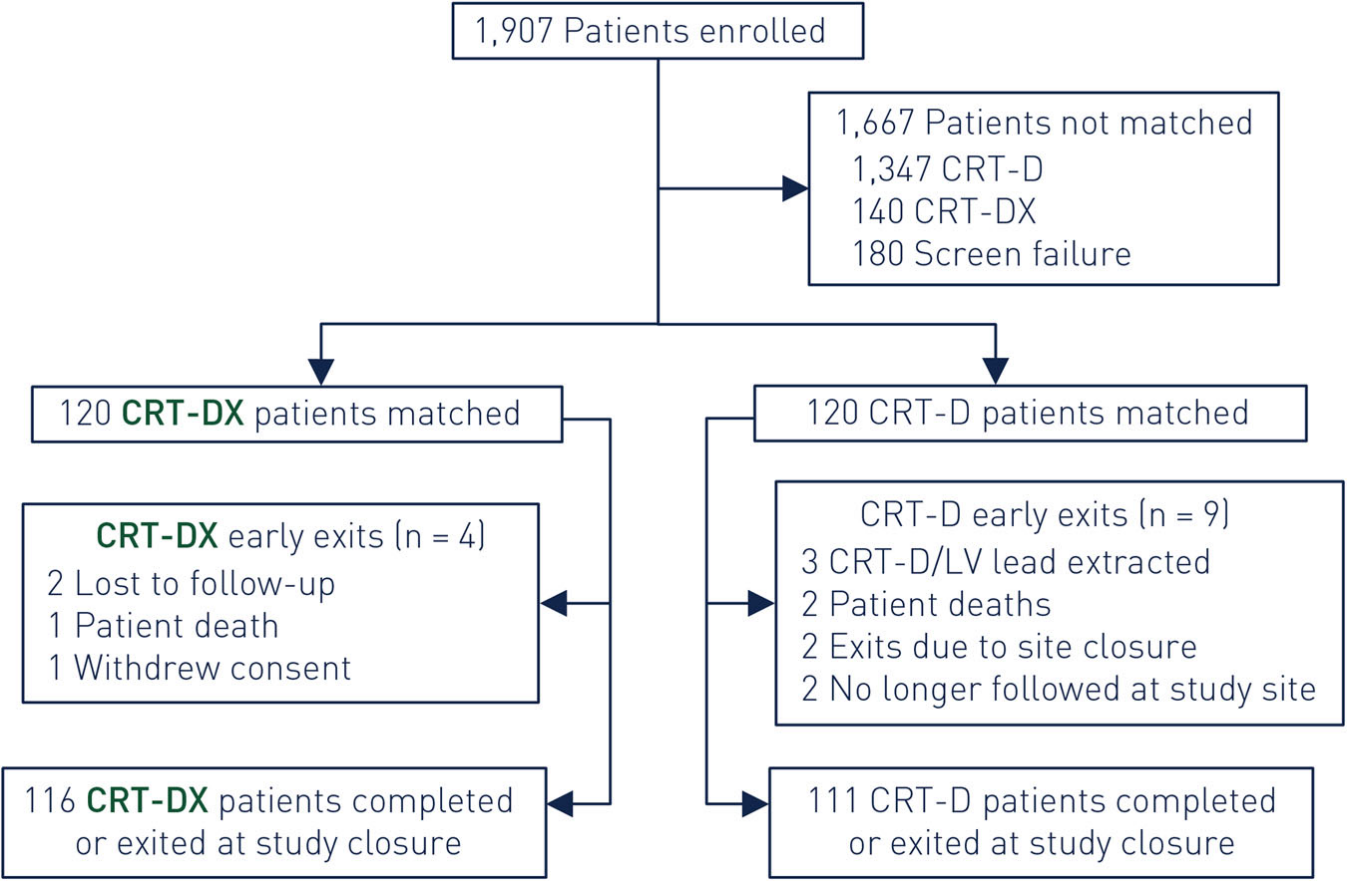
Study population flow chart. This flow chart shows the study population throughout follow‐up and distribution of matched subject pairs taken from the QP ExCELs study. CRT, cardiac resynchronization therapy; QP ExCEL, Sentus QP—Extended CRT Evaluation With Quadripolar Left Ventricular Lead

The goal of this subanalysis is to compare the overall complication rates and CRT responses in matched cohorts implanted with a two‐lead CRT‐DX and three‐lead CRT‐D ICD system (each CRT‐DX subject paired with a CRT‐D control). All subjects in this subanalysis were de novo implants (CRT‐DX: Intica 7 HF‐T QP; CRT‐D: Ilivia 7 HF‐T QP; BIOTRONIK SE & Co KG) and selected from the QP ExCELs general patient population with a minimum of 6 months of possible follow‐up time. In total, 120 matched pairs were identified using a 1 to 1 Greedy algorithm matched for gender, NYHA class, and heart failure etiology, plus an allowable age difference of ±8 years. These subjects were enrolled at 50 US study sites. A study flow diagram for this study population is provided in Figure 1.

Device diagnostic data, CRT responses, and complications were collected at required visits and via remote follow‐up utilizing daily transmissions from the Home Monitoring system (BIOTRONIK SE & Co KG) throughout the follow‐up period. NYHA class was collected at baseline and at the 6‐month follow‐up visit. A minimum of 10 daily transmissions were required to be included for analysis of device data. All device data was assessed by taking an average of all per subject means obtained for daily transmissions throughout follow‐up. LV pacing during CRT therapy is defined as the percentage of cardiac cycles that the LV is paced simultaneously with the RV, whether RV is paced or intrinsic. Subclinical AF in the form of device‐detected atrial high rate episode (AHRE) is nominally programmed as a counter of 36/48 atrial events more than 200 bpm, whereas AHRE burden is defined as the % of time the device has detected an atrial tachyarrhythmia. Each device is equipped with an accelerometer to measure subject activity % as a percentage of time the subject is physically active in a day. Device tachyarrhythmia settings and programming were determined per investigator discretion. All available shock therapies were adjudicated by two independent physicians.

### 2.1 Study primary endpoint

The primary endpoint was defined as the freedom from implanted system and implant‐related major complications. Major complications were defined as events related or possibly related to the implanted system or the implant procedure and requiring invasive intervention to resolve. All major complications were adjudicated by an independent Clinical Events Committee (CEC) comprised of physicians blinded to device cohort.

In addition, minor complications were evaluated for each cohort. Minor complications were defined as events related or possibly related to the implanted system or the implant procedure and not requiring invasive intervention to resolve.

### 2.2 Statistical analysis

Continuous variables were reported as means with standard deviation (SD) or as median with interquartile range (IQR) when normality was not met. Categorical variables were presented as frequencies with percentages. The cohorts were compared using a two‐tailed paired *t* test or Wilcoxon signed‐rank test for continuous variables, while McNemar's test, or Bowker's test were used for categorical variables. A Yates correction of 0.5 was used for continuity when zero cell counts were present. The 95% confidence intervals (CI) were calculated for the difference in continuous variables, as well as, for paired proportion differences. The major complication rates for the endpoint were evaluated using the Kaplan‐Meier curve with 95% CI based on the Peto SD. All statistical analyses were conducted using SAS 9.4 (SAS Institute, Cary, NC) with a significance level of 0.05.

## 3 RESULTS

Clinical characteristics of the study population at the time of enrollment are provided in Table 1. For this subanalysis, total follow‐up times were 152.2 and 165.7 subject‐years, with mean subject follow‐up times of 1.3 and 1.4 years for the CRT‐DX and CRT‐D cohorts, respectively.

**Table 1:** Clinical characteristics of the study population at enrollment

Variable	Matched cohort		*P* value
	CRT‐DX (n = 120)	CRT‐D (n = 120)	
Male, n (%)	83 (69.2%)	83 (69.2%)	‐‐‐‐‐^a^
Age, y	67.8 ± 10.93	67.8 ± 10.83	.9416
Body mass index, kg/m^2^	31.4 ± 8.35	29.5 ± 5.76	.0583
NYHA class I	1 (0.8%)	1 (0.8%)	‐‐‐‐‐^a^
NYHA class II	47 (39.2%)	47 (39.2%)	‐‐‐‐‐^a^
NYHA class III	71 (59.2%)	71 (59.2%)	‐‐‐‐‐^a^
LVEF, %	25.3 ± 6.46	25.5 ± 6.46	.8429
HF etiology, n (%)			
Ischemic	72 (60.0%)	72 (60.0%)	‐‐‐‐‐^a^
Nonischemic	48 (40.0%)	48 (40.0%)	‐‐‐‐‐^a^
ICD implant indication, n (%)			
Primary prevention	116 (96.7%)	111 (92.5%)	.1655
Secondary prevention	4 (3.3%)	9 (7.5%)	.1615
Electrocardiographic data			
Left bundle branch block, n (%)	76 (63.3%)	90 (75.0%)	.2482
Right bundle branch block, n (%)	9 (7.5%)	16 (13.3%)	.1615
AV block 1st, n (%)	11 (9.2%)	29 (24.2%)	.1113
AV block 2nd, n (%)	2 (1.7%)	2 (1.7%)	.1113
AV block 3rd, n (%)	2 (1.7%)	1 (0.8%)	.1113
QRS duration, ms	149.3 ± 25.36	157.5 ± 18.88	**.0075** *
Comorbidities, n (%)			
HTN	99 (82.5%)	90 (75.0%)	.1699
Diabetes	56 (46.7%)	52 (43.3%)	.5994
CAD	66 (55.0%)	65 (54.2%)	.8415
TIA/stroke	10 (8.3%)	9 (7.5%)	.8185
Valvular disease	32 (26.7%)	17 (14.2%)	**.0163** *
COPD	23 (19.2%)	14 (11.7%)	.1060
Medications, n (%)			
Diuretics	92 (76.7%)	79 (65.8%)	.0579
Beta blockers	109 (90.8%)	107 (89.2%)	.6547
Ca^++^ channel blockers	11 (9.2%)	12 (10.0%)	.8273
ACEi	53 (44.2%)	52 (43.3%)	.9013
ARB	38 (31.7%)	43 (35.8%)	.4838
Amiodarone	8 (6.7%)	11 (9.2%)	.4913
Digitalis	10 (8.3%)	3 (2.5%)	**.0348** *

*Note*: Values are given as mean ± SD or n (%) unless otherwise indicated.

Abbreviations: ACEi, ace inhibitor; ARB, angiotensin II receptor blocker; AV, atrioventricular; CAD, coronary artery disease; COPD, chronic obstructive pulmonary disease; CRT, cardiac resynchronization therapy; HF, heart failure; HTN, hypertension; ICD, implantable cardioverter‐defibrillator; LVEF, left ventricular ejection fraction; NYHA, New York Heart Association; SD, standard deviation; TIA, transient ischemic attack.

^a^ Clinical variables with a *P* value of “‐‐‐‐‐” represent an exact match between the cohorts.

* *P* values of less than .05 are in bold.

### 3.1 Primary endpoint analysis

**Figure 2:**
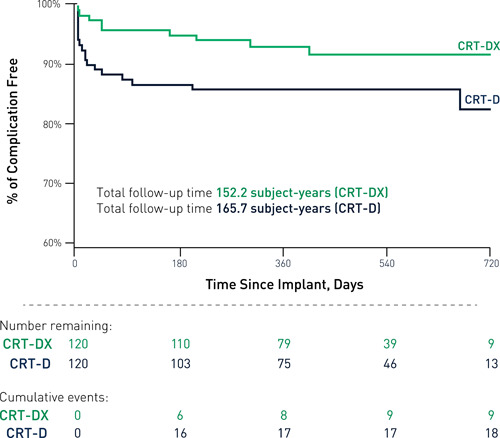
Freedom from primary endpoint major complications over total follow‐up. CRT, cardiac resynchronization therapy

To evaluate the incidence of major complications over follow‐up exposure postimplant, the calculated Kaplan‐Meier curve is provided in Figure 2. Among both cohorts, a total of 33 major complication events were adjudicated as related or possibly related to the implanted system or implant procedure over the follow‐up period.

Freedom from major complication was observed in 92.5% and 85.0% of subjects (*P* = .0495; 95% CI: 0.1%‐14.9%) in the CRT‐DX and CRT‐D cohorts, respectively. Nine subjects (7.5%) in the CRT‐DX cohort had a total of 11 major complications, compared with 18 subjects (15.0%) in the CRT‐D cohort with a total of 22 major complications. One event reported as a Staphylococcus bacteremia resolved with a total system extraction and was excluded from the CRT‐D cohort as the CEC adjudicated the event as a secondary infection.

Three subjects (2.5%) in the CRT‐DX cohort had an RV lead dislodgement event which required revision, compared with two subjects (1.7%) in the CRT‐D cohort. In addition, six subjects (5.0%) in the CRT‐D cohort had a total of six right atrial (RA) lead dislodgements which required revision. Five of the six RA lead dislodgments occurred within the first 45 days of follow‐up.

In the CRT‐DX cohort, no additional major complications were adjudicated as related or possibly related to the RV lead, and no subjects in the CRT‐DX cohort required an RA lead to be implanted over the follow‐up period. Sixteen subjects (13.3%) in the CRT‐DX cohort had a total of 18 minor complications, compared with 15 subjects (12.5%) in the CRT‐D cohort with a total of 17 minor complications. Twenty of 22 (90.9%) subjects with any major or minor LV lead‐related complication involving extracardiac stimulation were resolved with LV vector or other reprogramming. The two remaining subjects with extracardiac stimulation were resolved with electrical abandonment or replacement of the LV lead.

The percentage of subjects in each cohort with a major or minor complication related or possibly related to a specific system component (implant procedure, ICD device, RA lead, RV lead, LV lead) is provided in Table 2.

**Table 2:** Percentage of subjects with major/minor complication by system component

Reason for complication	Subjects with major complications		*P* value	Subjects with minor complications		*P* value
	CRT‐DX (n = 120)	CRT‐D (n = 120)		CRT‐DX (n = 120)	CRT‐D (n = 120)	
RA lead‐related						
Dislodgement	N/A	6, 5.0%	⋯	N/A	0, 0.0%	⋯
RV lead‐related						
Dislodgement	3, 2.5%	2, 1.7%	.8230	0, 0.0%	0, 0.0%	⋯
Extracardiac stimulation	0, 0.0%	0, 0.0%	⋯	1, 0.8%	0, 0.0%	.6171
LV lead‐related						
Dislodgement	5, 4.2%	8, 6.7%	.4510	0, 0.0%	0, 0.0%	⋯
Extracardiac stimulation	0, 0.0%	2, 1.7%	.2888	9, 7.5%	11, 9.2%	.7237
High impedance	1, 0.8%	0, 0.0%	.6171	4, 3.3%	0, 0.0%	.0801
Oversensing	1, 0.8%	0, 0.0%	.6171	0, 0.0%	0, 0.0%	⋯
Pulse generator related						
Inability to defibrillate	0, 0.0%	1, 0.8%	.6171	0, 0.0%	0, 0.0%	⋯
Electronic failure	1, 0.8%	0, 0.0%	.6171	0, 0.0%	0, 0.0%	⋯
Discomfort/pain	0, 0.0%	0, 0.0%	⋯	1, 0.8%	0, 0.0%	.6171
Implant related						
Pneumothorax	0, 0.0%	1, 0.8%	.6171	0, 0.0%	1, 0.8%	.6171
Pericardial effusion	0, 0.0%	1, 0.8%	.6171	0, 0.0%	0, 0.0%	⋯
Hematoma	0, 0.0%	1, 0.8%	.6171	0, 0.0%	0, 0.0%	⋯
Infection	0, 0.0%	0, 0.0%	⋯	0, 0.0%	3, 2.5%	.1489
Pleural effusion	0, 0.0%	0, 0.0%	⋯	1, 0.8%	0, 0.0%	.6171
Thrombosis	0, 0.0%	0, 0.0%	⋯	0, 0.0%	1, 0.8%	.6171
Arrhythmia	0, 0.0%	0, 0.0%	⋯	1, 0.8%	0, 0.0%	.6171

*Note*: A major complication is defined as events related or possibly related to the implanted system or the implant procedure and requiring invasive intervention to resolve. A minor complication is defined as events related or possibly related to the implanted system or the implant procedure and not requiring invasive intervention to resolve. A major/minor complication in more than one system component is possible; therefore, the total in Table 2 may be more than the number of subjects with one or more major/minor complication.

Abbreviation: CRT, cardiac resynchronization therapy.

### 3.2 Device‐detected CRT responses

Of the 120 matched subject pairs, 118 subjects in the CRT‐D cohort and 117 subjects in the CRT‐DX cohort met the minimum number of daily remote transmissions to analyze device diagnostic data. The average daily transmission rate was 88.1% and 91.1% for subjects in the CRT‐D and CRT‐DX cohorts, respectively.

Over the follow‐up period, the device‐detected median (IQR) LV pacing during CRT was similar for both cohorts at 98.4% (Q1: 95.4%, Q3: 99.8%) and 98.9% (Q1: 97.6%, Q3: 99.8%), in the CRT‐DX and CRT‐D cohorts, respectively (*P* = .2025; 95% CI:−0.6%‐0.2%). Device‐detected median daily subject activity % in the CRT‐DX cohort was 7.9% (Q1: 5.5%, Q3: 11.2%) compared with 8.6% (Q1: 5.7%, Q3: 12.1%) in the CRT‐D cohort (*P* = .1647; 95% CI:−2.0%‐0.9%).

To characterize the distribution of AF in each cohort over the follow‐up period, median daily AHRE burden and maximum daily AHRE burden episode duration grouped using cut‐offs of 6 minutes, 5.5 hours, and 24 hours are provided in Table 3.

**Table 3:** Distribution of device‐detected AHRE in each cohort

Cohort	0 min ≤ AHRE <6 min	6 min ≤ AHRE <5.5 h	5.5 h ≤ AHRE < 24 h	24 h	*P* value
Percentage of subjects with median daily AHRE burden sustained over 6 min, 5.5 h, and 24 h					
CRT‐DX cohort (n = 117)	89, 76.1%	6, 5.1%	10, 8.6%	12, 10.3%	**.0157** *
CRT‐D cohort (n = 118)	95, 80.5%	17, 14.4%	1, 0.9%	5, 4.4%	
Percentage of subjects with maximum daily AHRE episode duration sustained over 6 min, 5.5 h, and 24 h					
CRT‐DX cohort (n = 117)	79, 67.5%	9, 7.7%	7, 6.0%	22, 18.8%	.9685
CRT‐D cohort (n = 118)	76, 64.4%	13, 11.0%	9, 7.6%	20, 17.0%	

Abbreviations: AHRE, atrial high rate episode; CRT, cardiac resynchronization therapy.

* *P* values of less than .05 are in bold.

Device‐detected median daily atrial sensing amplitudes were stable and similar when compared between the two cohorts over the subanalysis follow‐up duration at 3.7 mV (Q1; 2.1, Q3: 6.0) and 3.6 mV (Q1: 2.7, Q3: 4.8) in the CRT‐DX and CRT‐D cohorts, respectively (*P* = .7835; 95% CI:−0.6%‐0.6%).

### 3.3 Clinical health status parameters

The total incidence of all‐cause mortality was 1.3%. Two subject deaths (1.7%) occurred in the CRT‐D cohort, and one subject death (0.8%) occurred in the CRT‐DX cohort over the follow‐up period. No cardiovascular deaths were noted. In addition, three subjects (2.5%) in the CRT‐DX cohort had a total of four heart failure (HF) hospitalization events, and three subjects (2.5%) in the CRT‐D group had a total of three HF hospitalization events. Two subjects in the CRT‐D cohort required a heart transplant procedure, compared to none in the CRT‐DX cohort.

**Figure 3:**
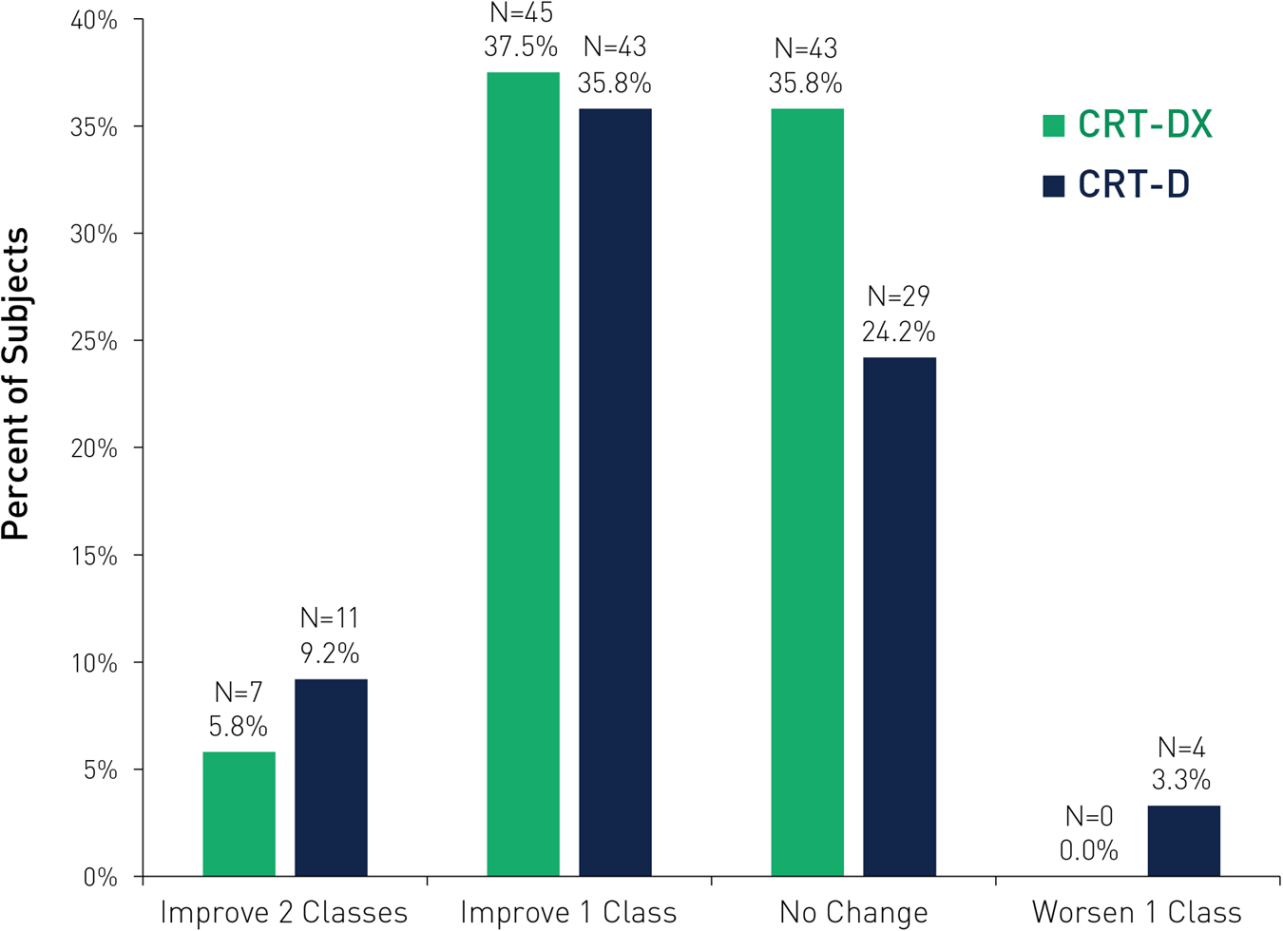
NYHA changes at 6 months follow‐up. An NYHA was not obtained at baseline and/or 6 months for 25 and 33 subjects in the CRT‐DX and CRT‐D cohorts. All percentages are displayed as absolute percentages (out of 120 subjects). CRT, cardiac resynchronization therapy; NYHA, New York Heart Association

When NYHA changes at 6 months were evaluated, improvements in NYHA class were 43.3% and 45.0% in the CRT‐DX and CRT‐D cohorts, respectively. In addition, four subjects in the CRT‐D cohort were documented with a worsened NYHA class. Among the 18 subjects in both cohorts with an NYHA improvement of two classes, 14 subjects (77.8%) had a nonischemic heart failure etiology at enrollment. Additionally, three of four subjects (75.0%) with an NYHA worsening had an ischemic heart failure etiology at enrollment. No subjects in either cohort had an NYHA worsen by two classes. NYHA changes at 6 months follow‐up are provided in Figure 3. Due to NYHA data not being obtained for all subjects, inferential analysis would exclude up to 40% of the matched cohort (for each subject with a missing NYHA, the pair would also be excluded); therefore, *P* values for changes in NYHA were not calculated.

### 3.4 Defibrillator therapy during follow‐up

Throughout the follow‐up, 24 appropriate shock therapy events occurred in eight subjects (6.7%) in the CRT‐D cohort, compared with four shock therapy events which occurred in three subjects (3.3%) in the CRT‐DX cohort. A total of seven CRT‐D subjects (5.8%) experienced at least one inappropriate shock vs one subject with a CRT‐DX (0.8%). Regular supraventricular tachyarrhythmias or sinus tachycardia was the most common reason for inappropriate therapy (CRT‐DX, one subject; CRT‐D, four subjects). Other causes were oversensing due to lead dislodgement (CRT‐D, one subject), oversensing due to unknown cause (CRT‐D, one subject), and electromagnetic interference (CRT‐D, one subject).

## 4 DISCUSSION

Previously reported CRT system complication rates vary considerably, with some studies reporting overall rates of complications requiring intervention of approximately 3% to 7%,^7^, ^16^, ^17^ and additional studies reporting higher complication rates of approximately 10% to 11%.^4^, ^18^, ^19^ Lead revisions vary from 1.3% to 10.0% for subjects implanted with a CRT‐D system and make up a large portion of the overall complication rate.^4^, ^7^, ^16^, ^17^, ^18^ These variations make meaningful comparisons of complication rates difficult, and are in part due to differences in follow‐up, a lack of standardized definition for a complication event, study design variations, advancements in technology used and differences in reporting accuracy. In addition, gender, device type, implant center and operator volume, the presence of AF/flutter, and advanced heart failure are all important predictors which can affect complication rates within a patient population.^5^, ^7^, ^20^, ^21^

This is the first comparison of complication rates between subjects implanted with two‐lead CRT‐DX and three‐lead CRT‐D devices. The percentage of subjects with one or more major complication in the total study population was 11.3%. Half as many subjects in the CRT‐DX cohort had one or more major complications compared to the CRT‐D cohort, representing an absolute 7.5% lower rate of complication in the CRT‐DX cohort. Major complications were more often associated with the LV lead in both cohorts. However, the higher rate of major complication for the CRT‐D cohort was driven primarily by RA lead dislodgements. In particular, there were six subjects (5%) with an RA lead dislodgment in the CRT‐D cohort. This rate is higher when compared to CRT system complication rates for RA leads of 1.3% to 3.5% seen in other studies,^7^, ^18^ although at least one study reported atrial lead dislodgments in 4% of subjects implanted with a dual‐chamber ICD.^15^

To further interpret these findings, RA lead dislodgments and overall complication rates in the CRT‐D cohort were compared with a larger cohort comprised of the remaining subjects in the QP ExCELs registry with a minimum of 6 months of follow‐up time (excludes the subjects in the 120 subject CRT‐D cohort). In total, 1094 subjects were evaluated over a mean follow‐up time of 2.1 years. A lower rate of RA lead dislodgements (2.3%) and overall major complications (11.5%) was observed in the 1094 subject CRT‐D cohort compared to the 120 subject CRT‐D cohort. Specific reasons for the differences in complication rates between the CRT‐D cohorts of 120 subjects and 1094 subjects are not clearly understood.

Overall, major complications were slightly higher when compared to prior studies; therefore, the *χ* ^2^ test was used to evaluate possible associations between gender or site enrollment counts and major complication rates in the 1094 subject CRT‐D cohort. A statistically significant association between female gender and major complications was observed (*P* = .0404) and is consistent with findings in other studies.^21^ The QP ExCELs registry enrolled a higher percentage of females (30.8% female reflected in the CRT‐DX and CRT‐D cohorts) compared to other CRT‐D studies which may explain the increased rates of major complications in this analysis. No meaningful association was observed between site enrollment counts and major complications.

Numerous prior publications have demonstrated an association between higher pacing and favorable patient outcomes, with a goal of pacing the ventricles as close to 100% as possible.^22^, ^23^, ^24^ Moreover, AF, atrial tachyarrhythmia, and ventricular ectopy have been identified as potential causes of reduced ventricular pacing during CRT.^25^ Our data show similar device‐detected medians of LV pacing during CRT of 98.4% and 98.9% in the CRT‐DX and CRT‐D cohorts, respectively. A slightly higher variation in the percent of pacing was observed in the CRT‐DX cohort, as evidenced by IQR values of 4.4% with CRT‐DX vs 2.2% with CRT‐D. The relationship between ventricular pacing during CRT and atrial arrhythmia burden can vary based on several factors including conduction system characteristics and programmed device settings, making direct associations between these two variables difficult; however, the increased variation in the ventricular pacing percentage seen in the CRT‐DX cohort reflects the higher daily median AHRE burden in this cohort (Table 3).

One previous study prospectively compared CRT performance and physiologic responses in a group of heart failure subjects implanted with a CRT‐DX and CRT‐D system and found no differences in LV reverse modeling, cardiopulmonary exercise performance, and NYHA class improvement.^13^ In addition, several studies have demonstrated the utility of the DX lead.^11^, ^12^, ^26^ Specifically, it was shown in a cohort of 249 subjects that P‐wave amplitudes in the DX ICD system cohort were comparable and stable (~3.5 mV), and that SVT discrimination was equivalent when compared to a dual‐chamber ICD system over 12 months of follow‐up.^15^ Moreover, the SENSE trial showed comparable rates of subclinical AF in the DX system compared to a dual‐chamber ICD system, with no incidence of inappropriate therapies delivered in the DX ICD system cohort over 1 year of follow‐up.^10^ In this subanalysis on CRT‐DX, we saw similar low rates of inappropriate shock with only one subject in the CRT‐DX cohort receiving inappropriate shock during the 1.3 years of follow‐up.

A higher number of appropriate shock therapy events were observed in the CRT‐D cohort compared to the CRT‐DX cohort. A definitive explanation for this difference in appropriate shocks is uncertain but it is hypothesized that it could be explained by differences in specific clinical parameters between the cohorts. There were more subjects with a secondary prevention indication for defibrillator therapy in the CRT‐D cohort (7.5%) compared to the CRT‐DX cohort (3.3%), and there was a statistically significant wider QRS duration in the CRT‐D cohort compared with the CRT‐DX cohort (*P* = .0075). In addition, tachyarrhythmia settings were determined per physician discretion, and were not standardized between the cohorts. This lack of homogeneity between specific clinical parameters between the two cohorts might explain this finding.

Overall, our subanalysis results show significantly fewer complications and lower rates of inappropriate shock in the CRT‐DX cohort, while supporting similar health status outcomes with regards to patient mortality, heart failure hospitalizations, and NYHA class improvement as compared to a CRT‐D cohort.

### 4.1 Limitations

Our study has several limitations. While the data were collected from a large, prospective study, this subanalysis was conducted retrospectively and endpoints were not predefined. In contrast to many large, national registry‐based studies that rely only on site identification and reporting of events, this study utilized frequent monitoring and careful review of source documentation plus independent adjudication to ensure accuracy and limit underreporting. This may have resulted in a higher rate of reported complications. Device programming, including pacing modes, ventricular tracking rates, and tachyarrhythmia settings, were not controlled for in this study and were determined per physician discretion. Last, diagnosis of AF history at enrollment was not collected as part of the QP ExCELs study.

## 5 CONCLUSIONS

Our subanalysis results show the CRT‐DX system can provide similar CRT responses and significantly fewer complications, indicating that the CRT‐DX system is a capable alternative in patients without an atrial pacing indication.

## Data Availability

The data that support the findings of this study are available upon reasonable request. The data are not publicly available due to privacy restrictions.
